# The Impact of Number of Medications on Falls in Aging Persons with Human Immunodeficiency Virus

**DOI:** 10.3390/life13091848

**Published:** 2023-08-31

**Authors:** Leanne W. Thai, Lucas Hill, Shannon Balcombe, Afsana Karim, Maile Young Karris

**Affiliations:** 1 Department of Pharmacy, Scripps Mercy Hospital, San Diego, CA 92103, USA; 2 Department of Pharmacy, University of California San Diego, San Diego, CA 92103, USA; 3 Department of Medicine, University of California San Diego, San Diego, CA 92103, USA

**Keywords:** polypharmacy, falls, aging, HIV

## Abstract

We aimed to evaluate the impact of polypharmacy on the risk of having a fall in older persons with HIV (PWH). PWH at least 50 years of age who were seen at our institution from September 2012 to August 2017 were included. Unique participants were selected for either a case or control cohort depending on the presence of a documented fall during the study time period. Demographics, HIV-related measures, VACS score, number of medications, as well as the impact of taking benzodiazepines and opioids were compared between the two cohorts. Fall was documented for 637 patients compared to 1534 without a fall during the same time period. Multivariable logistic regression revealed that the total number of medications, having a higher VACS score, taking an opioid, being female sex assigned at birth, and having a lower nadir CD4 count were significantly associated with higher odds of having a fall. In this cohort of older PWH, taking a higher number of non-ARV medications significantly increased the odds of having a fall. In addition, taking an opioid resulted in the highest odds of having a fall. These results suggest the importance of deprescribing and addressing opioid use in reducing the risk of having a fall in older PWH.

## 1. Introduction

Persons with HIV (PWH) develop age-associated medical comorbidities earlier, and in excess to, their HIV-negative peers [1]. It is currently estimated that in the U.S., by 2030, 75% of PWH will be 50 years or older, and almost 30% will have three or more age-related illnesses [2]. This aging epidemic is largely the result of effective antiretroviral (ARV) treatments that prolong the lifespan of PWH [1]. While HIV-associated morbidity has decreased over time, morbidity related to other chronic medical conditions is increasing. In addition to combination antiretroviral therapy (ART), which usually consists of three distinct antiviral medications, PWH are taking more medications to manage multi-morbidity and are increasingly at risk for polypharmacy [2,3]. A recent retrospective study found that 58% of PWH aged 50–64 years took five or more prescription medications, in addition to ART [4].

The negative health impacts of polypharmacy (most commonly defined in the literature as ≥5 medications) include increased risks for drug interactions, serious adverse drug events, medical costs, and suboptimal medication adherence [5,6]. Clinical consequences are most pronounced in the elderly due to age-related changes in pharmacokinetics and pharmacodynamics. In the elderly, polypharmacy negatively impacts cognitive (delirium) and physical capabilities, and increases morbidity and mortality [7,8,9]. Specifically, polypharmacy in older adults results in a decreased physical functioning, the inability to perform instrumental activities of daily living, and an increased risk of falls [10,11,12].

To date, the work on polypharmacy in older PWH has largely described the problem and extrapolated potential clinical consequences from the geriatric literature. Polypharmacy (not counting ARVs) appears more common in older PWH compared to HIV-seronegative persons [13]. In a retrospective study, 81% of PWH were prescribed at least one ARV/non-ARV combination that was contraindicated or had potential for a clinically significant interaction [14]. A cross-sectional study of an older cohort of PWH reported that at least one potentially inappropriate medication was prescribed in 52% of participants, 70% had at least one Category D (consider therapy modification) drug interaction, and 11% had a Category X (avoid combination) interaction [15]. A recent review of several studies assessing the risk of falls in PWH identified a frequency of 12% to 41% for any fall in a time period of the prior 6 to 24 months, depending on the study, with some studies identifying polypharmacy as a risk factor for having a fall. However, the study populations and types of analysis varied among the different studies included [16]. This study aims to build on the existing literature on polypharmacy in older PWH by exploring the association between the numbers of medications and a clinical consequence that has a high risk of significant morbidity: falls.

## 2. Methods

### 2.1. Study Population and Design

This study is a retrospective, single-site analysis of older PWH. The institutional review board at the University of California, San Diego (UCSD) approved this minimal risk study prior to implementation. Subjects at least 50 years of age with documented HIV-seropositive status with a clinic visit within our institution between September 2012 and August 2017 were included. All data were collected via the electronic medical record (Epic). Subjects were then divided into two groups, those with a documented fall, and those that did not have a documented fall. Documentation of fall within 6 months is a standard part of intake at each clinic visit. Exclusion criteria included the diagnosis of paraplegia or use of an assistive walking device, such as a cane, walker, or wheelchair.

### 2.2. Demographic and Medication Factors

Demographic data collected included: Age, sex assigned at birth (as documented in the medical record, which may not match with gender identity), race, and ethnicity. HIV-related factors included CD4+ T cell count, HIV viral load, HIV risk factor, CD4+ T cell nadir, and duration of HIV infection. The total number of non-ART medications was captured along with active prescriptions for an opioid or benzodiazepine medication, as well as number of ARV medications. The Veterans Aging Cohort Study (VACS) index was also calculated for each participant to characterize severity of comorbidities. Data were captured at the time of documentation of a fall or at the latest time point in the study period for non-fall controls. We did not pursue time-matched negative fall controls because the study time frame was performed in the modern ART era. 

### 2.3. Statistical Analyses

Characteristics of the two cohorts were compared using chi-square for categorical variables and unpaired Student’s t-test or Mann–Whitney U test for continuous variables. For race, ethnicity, and HIV risk factor, analysis was performed using a chi-square contingency table. For the primary endpoint of fall, a univariate analysis for predictors of falls was performed. Multivariable logistic regression was performed to assess variables associated with increased odds of having a fall, and included age, sex assigned at birth, race, ethnicity, CD4+ cell count, HIV viral load, nadir CD4+ cell count, time since diagnosis, VACS score, number of non-ARV medications, the presence of polypharmacy, and the presence of a benzodiazepine or opioid prescription in the models. Data were analyzed using MedCalc Version 22.009.

## 3. Results

### 3.1. Study Population

There were 2171 patients who met the inclusion criteria during the study period, of which 637 (29.3%) reported a fall in the previous 6 months. There was no statistically significant difference between the two cohorts based on race or ethnicity. Persons with reported falls were more likely to be female assigned at birth (16.8% vs. 10.4%, *p* < 0.0001) and were slightly older in age. No statistically significant differences were noted between the two cohorts based on the presence of detectable HIV viral load at the time of fall or median CD4+ T cell count. Persons who fell did have a slightly longer duration of HIV infection, lower nadir CD4+ T cell counts, a higher VACS index, and were more likely to have intravenous substance use as a risk factor for HIV (Table 1). After conducting multivariable logistic regression, being female assigned at birth was significantly associated with an increased risk of fall (*p* = 0.016, odds ratio (OR) 1.42 (1.07–1.90)). Having a higher VACS score (*p* < 0.0001, OR 1.025 (1.02–1.03)) was also significantly associated with having a fall, as was lower CD4+ nadir (*p* = 0.002).

### 3.2. Medication-Related Factors That Contribute to Risk of Fall

To evaluate the impact of polypharmacy on falls, we evaluated differences in total non-ARV medications and prescribed benzodiazepine and opioid use between persons with a fall and those without a fall. Persons with a fall were prescribed significantly more non-ARV medications than persons who did not fall (mean 11.2 vs. 7.5, *p* < 0.0001) and were more often diagnosed with polypharmacy. Opioid prescriptions were also more common in persons with a fall compared to those who did not fall, whereas there was no significant difference in the frequency of benzodiazepine use (Table 2). After completing multivariable logistic regression, a higher number of non-ART medications increased odds of falling, with each additional medication increasing the odds of fall by 7% (*p* < 0.0001, OR 1.07 (1.05–1.09)). Having an opioid prescription resulted in the greatest odds of falling, resulting in over a 2-fold higher risk (*p* < 0.0001, OR 2.10 (1.69–2.60)).

## 4. Discussion

This retrospective study demonstrates that female sex assigned at birth, disease severity (VACS), total number of non-ARV medications, and prescription opioids all increase the odds of falls in older PWH. Falls is a common clinical consequence of polypharmacy in older adults [17]. Falls may result in serious injury, and if accompanied by hip fracture, significant resultant morbidity and mortality [18,19]. In this study, we aimed to determine if polypharmacy contributes to the risk of falls in a population of chronologically young, but older PWH (age 50 years and older). This study is consistent with a prior publication associating polypharmacy and opioid use as risk factors for falls in a population of PWH between the ages of 45–65 [17]. Our population was older, but a lower proportion reported falls (29% compared to 48%). This is likely related to study differences in the characterization of falls (6 months compared to 12 months). Unlike this previous study, we found the VACS index was associated with increased odds of falls. Of note, VACS scores were higher in both arms of our study compared to previous work, and could be due to differences in the disease severity of the different populations reflective of the differences in age. This highlights the heterogeneity of older PWH. In addition, our study confirmed the findings of previous studies that females assigned at birth with HIV are at an increased risk of having a fall [17].

In our cohort, having polypharmacy (defined as greater than or equal to five non-ARV medications) or not was less useful than the total number of medications as a falls predictor. This could be due to statistical differences of power between evaluating categorical versus continuous measures [20]. However, while the definition of polypharmacy as five or more medications is the most frequently used definition, it is arbitrary and varies widely among different studies [21]. Our results may also suggest that five or more medications is not the ideal definition of polypharmacy in older PWH, and the more important observation is that the odds of fall increases with each additional medication. We also observed that CD4+ T cell count, the duration of HIV infection, and detectable HIV viremia did not significantly increase the odds of a fall in the multivariable models, suggesting a minimal “HIV effect” on fall risk. However, having a lower CD4+ count nadir was associated with increased odds of having a fall. These observations are somewhat in line with a previously published paper on the VACS (both PWH and HIV-seronegative controls), demonstrating a dose–response risk of hospitalization and mortality associated with non-ART medication use that was independent of disease severity or HIV status [7]. A previous study by Erlandson et al. evaluating predictors of falls in those with HIV or those at risk of HIV did not find any significant difference in the risk of falls based on HIV serostatus. However, in those living with HIV, the number of medications was associated with an increased risk of fall [22]. In addition, modern antiretroviral therapy now provides various options of 2-drug versus 3-drug antiretroviral regimens, allowing for a reduction in the number of medications being taken in PWH. Although our study, as well as previous studies, excluded antiretroviral therapy when determining the number of medications or presence of polypharmacy, the impact of antiretroviral regimens with fewer medications on the impact of falls or other significant adverse effects in older PWH should be considered for future research [13,22]. Prescription opioids increased the odds of a fall over 2-fold in older PWH. Chronic pain is very common in older PWH, and PWH are more likely to be prescribed opioids, and at higher morphine milligram equivalents of opioids, than HIV-seronegative persons [23,24,25,26]. In older adults, non-opioid medications used for the management of pain are often contraindicated due to risk of severe adverse events, and thus, opioids are commonly used [27]. The finding that opioids specifically increase fall risk in older PWH should be considered in discussions about unintended consequences of opioid use in older PWH. These findings provide further evidence that novel approaches to pain management are necessary, and opioid deprescribing should be a priority in this population.

There are several limitations to our study. One limitation was that data extraction was limited to what was available in the EMR, and was based on the accuracy of the EMR user. Thus, active medication lists are susceptible to both missing medications as well as an overestimation of the number of medications a patient is actually taking. This also limited the assessment of opioid or benzodiazepine use to medication lists [28]. In addition, although the presence of viral suppression was evaluated, specific antiretroviral regimens were not documented. It is known that various antiretroviral regimens, particularly boosted protease inhibitor-based regimens, may interact with opioids and increase the risk of adverse effects, and the impact of these drug interactions on outcomes in our population should be considered when evaluating for fall risk [29]. It should also be noted that the start of our study period was 2012, and newer antiretroviral regimens approved since the start of the study period in general may have improved safety profiles, including in older PWH. Second, the study design did not allow for the quantitative capture of measures such as frailty, neuropathy, or other physical factors that are associated with fall risk, as previous studies have identified frailty as well as imbalance symptoms as being associated with an increased risk of falls in PWH [22,30]. The study was conducted at only one site, and although the demographics are similar to many inter-city HIV clinics, this limits the generalizability of the results. This study limited medication evaluations to benzodiazepines and opioids, and future work should consider other fall risk-inducing drugs (drugs for cardiovascular disease, antidepressants, urological spasmodics, etc.) [31,32]. Additionally, because we excluded persons at high risk for fall at baseline (paraplegia, walking device) we cannot extrapolate our findings to this highly vulnerable population. Finally, fall was the only clinical outcome measured in this study and other clinically relevant outcomes of polypharmacy will require ongoing research.

## 5. Conclusions

This study demonstrates the impact of the number of medications, disease severity, and specific medication classes (i.e., opioids) on the odds of falls in older PWH. This adds to the current literature, describing the extent and details of polypharmacy in older PWH. The confirmation that polypharmacy contributes to falls in older PWH further provides evidence supporting deprescription in this population [33].

## Figures and Tables

**Table 1:** Analysis of demographics and HIV characteristics.

	Fall (n = 637)	No Fall (n = 1534)	*p*-Value
**Median Age (IQR)**	58 (54–63)	56 (53–61)	<0.00001
**Sex Assigned a Birth (%)** Female	107 (16.8)	159 (10.4)	<0.0001
**Race (%)**			0.20
White	402 (63.1)	945 (61.6)	
Black	101 (15.9)	207 (13.5)	
Asian	8 (1.3)	34 (2.2)	
Other	119 (18.7)	313 (20.4)	
Unknown	7 (1.1)	35 (2.3)	
**Ethnicity (%)**			0.12
Hispanic	133 (20.9)	327 (21.3)	
Non-Hispanic	493 (77.4)	1156 (75.4)	
Other/Unknown	11 (1.7)	51 (3.3)	
**Median CD4+ T cell count (IQR)** (cells/mm^3^)	494 (295–732)	507 (322–705)	0.66
**Detectable HIV viral load (%)** (>50 copies/mL)	156 (24.5)	373 (24.3)	0.93
**HIV Risk Factor (%)**			<0.0001
MSM only	366 (57.5)	1045 (68.1)	
Any IVDU	113 (17.7)	163 (10.6)	
Heterosexual	133 (20.9)	258 (16.8)	
Transfusion	6 (0.9)	15 (1.0)	
Hemophilia	2 (0.3)	4 (0.3)	
Other/unknown	17 (2.7)	49 (3.2)	
**Median CD4+ T cell nadir (IQR)** (cells/mm^3^)	166 (58–324)	217 (72–389)	<0.001
**Median time since diagnosis (IQR)** (years)	21.1 (13.6–28.4)	19.6 (12.4–26.3)	0.002
**Median VACS score** (IQR)	32 (22–46)	25.5 (18–37)	<0.0001

**Table 2:** Analysis of medication-related variables.

	Fall (n = 637)	No Fall (n = 1534)	*p*-Value
**Mean number of medications** * (IQR)	11.2 (10.6–11.7)	7.5 (7.2–7.8)	<0.0001
**Number with polypharmacy** ** (%)	519 (81.5)	1001 (65.3)	<0.0001
**Number taking benzodiazepine** (%)	92 (14.4)	218 (14.2)	0.89
**Number taking opioid** (%)	290 (45.5)	348 (22.7)	<0.0001

* Non-ARV medications; ** Defined as ≥5 non-ARV medications.

## Data Availability

The data used to generate results of this study are not publicly available but can be made available upon reasonable request to the corresponding author.
